# Highly Responsive Chitosan-Co-Poly (MAA) Nanomatrices through Cross-Linking Polymerization for Solubility Improvement

**DOI:** 10.3390/gels8030196

**Published:** 2022-03-21

**Authors:** Anam Saleem, Naveed Akhtar, Muhammad Usman Minhas, Arshad Mahmood, Kifayat Ullah Khan, Orva Abdullah

**Affiliations:** 1 Department of Pharmaceutics, Faculty of Pharmacy, The Islamia University of Bahawalpur, Bahawalpur 63100, Pakistan; anamsaleempharmacist@gmail.com (A.S.); naveed.akhtar@iub.edu.pk (N.A.); 2 College of Pharmacy, University of Sargodha, Sargodha 40100, Pakistan; 3 College of Pharmacy, Abu Dhabi Campus, Al Ain University, Abu Dhabi 51133, United Arab Emirates; arshad.mahmood@aau.ac.ae; 4 Quaid-e-Azam College of Pharmacy, Sahiwal 57000, Pakistan; kifayat.rph@yahoo.com; 5 Hamdard Institute of Pharmaceutical Science, Hamdard University Islamabad, Islamabad 45600, Pakistan; orva_abdullah@yahoo.com

**Keywords:** nanomatrices, solubility enhancement, simvastatin, chitosan, toxicological evaluation

## Abstract

In this study, we report the highly responsive chitosan-based chemically cross-linked nanomatrices, a nano-version of hydrogels developed through modified polymerization reaction for solubility improvement of poorly soluble drug simvastatin. The developed nanomatrices were characterized for solubilization efficiency, swelling studies, sol-gel analysis, in vitro drug release studies, DSC, FTIR, XRD, SEM, particle size analysis, and stability studies. An in vivo acute toxicity study was conducted on female Winstor rats, the result of which endorsed the safety and biocompatibility of the system. A porous and fluffy structure was observed under SEM analysis, which supports the great swelling tendency of the system that further governs the in vitro drug release. Zeta sizer analyzed the particle size in the range of 227.8 ± 17.8 nm. Nano sizing and grafting of hydrophilic excipients to the nanomatrices system explains this shift of trend towards the enhancement of solubilization efficiency, and, furthermore, the XRD results confirmed the amorphous nature of the system. FTIR and DSC analysis confirmed the successful grafting and stability to the system. The developed nanomatrices enhanced the release characteristics and solubility of simvastatin significantly and could be an effective technique for solubility and bioavailability enhancement of other BCS class-II drugs. Due to enhanced solubility, efficient method of preparation, excellent physico-chemical features, and rapid and high dissolution and bio-compatibility, the developed nanomatrices may be a promising approach for oral delivery of hydrophobic drugs.

## 1. Introduction

Limited aqueous solubility is the major hurdle in the development of a drug delivery system [1]. A total of 70–90% of new chemical entities (NCEs) that are in the pipeline of pharmaceutical development are suffering from poor aqueous solubility [2,3,4,5,6]. The absorption a of drug in the gastro-intestinal tract (GIT) is limited by inadequate solubility and permeability [7]. Poor soluble drugs require high doses to reach the systemic circulation in order to execute clinical response [8]. To overcome this issue, in the current study, a highly porous and stimuli responsive, cross-linked nanomatrices drug delivery system was synthesized to enhance the solubility of poorly soluble drugs.

Simvastatin is obtained by fermentation of *Aspergillum terreus*, which is a fungi, in the form of inactive lactone, which is further hydrolyzed to active β-hydroxy acid and works as an anti-hyperlipidemic agent by inhibiting HMG-CoA reductase enzyme, which has been well-established in a human clinical trial [9]. Simvastatin is among the most potent statin and has been listed among the top five frequently prescribed medicines for reducing high cholesterol [10,11]. Apart from its role as anti-hyperlipidemic agent, its pleiotropic effects opens a totally new horizon in medical sciences, suggesting its anti-tumor [12], anti-cancer [13], cardio-protective [14], anti-inflammatory, immune-modulatory, and anti-diabetic potential [15]. Despite of its growing clamor as a pleotropic agent and established reputation for treating cardio-vascular disorders, its poor aqueous solubility restrains the absorption of simvastatin from GIT, and hence the drug becomes less bioavailable [16]. Many approaches have been reported to enhance the solubility of simvastatin, such as mechano-chemical complexation with polysaccharides like arabinogalactan [17], co-crystal with citric acid [18], encapsulation with low viscosity grade HPMC [19], nano-crystal technologies, nanogels/nanomatrices, nano-suspension, inclusion complex formation-based techniques, solid-lipid nanoparticles, liposomes, nano-capsule, and co-solvency [20,21,22,23,24,25].

Hydrogels are three-dimensional (3D) cross-linked polymeric chains with a high water-absorption capacity [26]. They can be employed as very efficient, stimuli responsive and biodegradable drug carriers in drug delivery systems [27,28]. Based upon the size of the obtained particles, hydrogels can be classified as macrogels, microgels, and nanogels/nanomatrices. Hydrogel particles fabricated in the nanometer range are termed as nanogels/nanomatrices [29], particles obtained in the micrometer range are termed as microgels, and particles bigger than micrometer range are termed as macrogels [30].

Herein, we report nanomatrices, a nano-version of hydrogels, as an emerging technique used for solubility improvement of poorly soluble drugs. Cross-linked nanomatrices refer to a three-dimensional cross-linked nano-sized polymeric network that is bio-compatible, amorphous, a soft biomaterial, and a suitable carrier for hydrophobic drugs [31,32,33,34]. Previously, Badshah et al. reported nanomatrices synthesized by crosslinking β-cyclodextrin with acrylic acid to enhance the solubility of chlorthalidone [25]. Khan et al. increased the solubility of olanzapine by synthesizing nanomatrices by cross-linking polaxamer-407 and AMPS [35]. Khalid et al. cross-linked hydroxypropyl β-cyclodextrin with AMPS for dissolution enhancement of dexibuprofen [36].

Chitosan is a non-toxic, linear hydrophilic cationic polymer of d-glucosamine that is degraded by colon micro-flora and is commonly reported for solubility enhancement of poorly soluble drugs [37,38,39]. Polysaccharides and proteins are the main stream candidates for developing nanomatrices drug delivery system owing to their bio-degradable, hydrophilic, and bio-compatible nature [40]. Film-forming ability, gelatinous features, bio-adhesion, and skin penetration-enhancing effects have all been enlisted as properties of chitosan. Chitosan may interact with negatively charged molecules and polymers due to its polymeric cationic properties [41].

Methacrylic acid is an α, β-unsaturated monocarboxylic acid that is an acrylic acid with a methyl group substituting for hydrogen at position 2, derived from acrylic acid, and is an acid conjugate of methacrylate. PH sensitivity and a high degree of swelling in basic solutions and collapsing in acidic solutions characterize the methacrylic acid polymer. Electrostatic ion repulsion between carboxylic acid and ions in buffered solutions causes this behavior [42]. Benzoyl peroxide is an oxidizer used for initiation of polymerization reaction by itself fragmenting into two free radical initiator and is preferred due to low cost, ease of use, and safety in method of polymerization [43]. *N*,*N*′-methylene bisacrylamide (MBA) is a bisacrylamide containing two terminal vinyl groups that can react with radicals and act as a cross-linker in polymerization reaction. It is preferred for its hydrophilicity and degree of cross-linking [44].

In the present work, the nanomatrices drug delivery system is designed and characterized to increase the solubility and dissolution profile of BCS class-II drug simvastatin by cross-linking hydrophilic polymer chitosan with methacrylic acid (MAA) using MBA as the cross-linker and benzoyl peroxide (BPO) as an initiator.

## 2. Results and Discussion

### 2.1. Sol-Gel Analysis and Stability Studies

**Figure 1:**
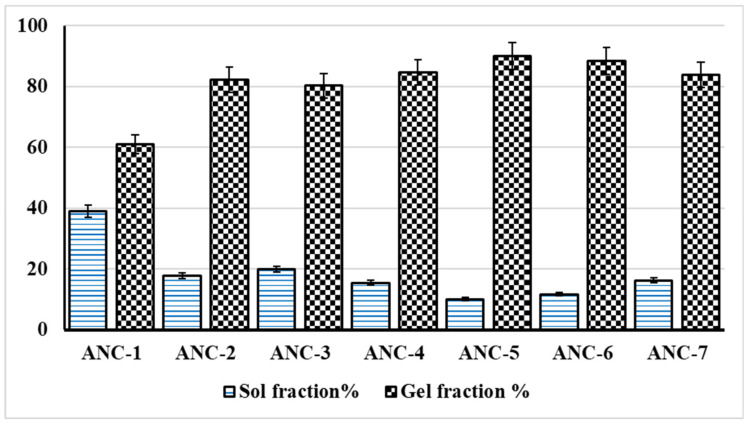
Sol-gel analysis of developed nanomatrices (ANC-1 to ANC-7) formulations.

The cross-linked and uncross-linked fraction of the polymeric network were determined by sol-gel analysis. By increasing the polymer (chitosan) concentration, we observed increased gelling fraction up-to a certain limit, i.e., ANC-1 to ANC-2, as shown in Figure 1. A further increase in the concentration of polymer resulted in decreased gelling, i.e., ANC-2 to ANC-3. This pattern suggested less tendency of the system to cross-link at this concentration, which may be due to insufficient quantity of monomer or cross-linker present in the system. Increase in the concentration of monomer, i.e., ANC-2, ANC-4, to ANC-5 we observed an increased gelling fraction, which may be due to the more available active sites for polymerization reaction, which increased the rate and extent of polymerization reaction. Badshah et al. have reported similar findings [25].

Increased concentration of cross-linker (MBA) (ANC-6) depicted an increase in gelling fraction due to the enhanced cross-linking potential of the system, which is consistent with the findings of Mahmood et al. [45]. However, with a further increase in cross-linker concentration (ANC-7), a decrease in gel fraction was observed due to the formation of tight cross-linkages that restricted the movement of polymeric chains, as shown in Figure 1 [46]. The formulation of simvastatin-loaded cross-linked nanomatrices remained stable during the specified study period, according to the stability investigations, as shown in Table 1. Physical appearance, percent encapsulation efficiency (%EE), FTIR spectra, and solubilization efficiency showed no significant changes, indicating that cross-linked nanomatrices are stable.

### 2.2. Swelling Studies

**Figure 2:**
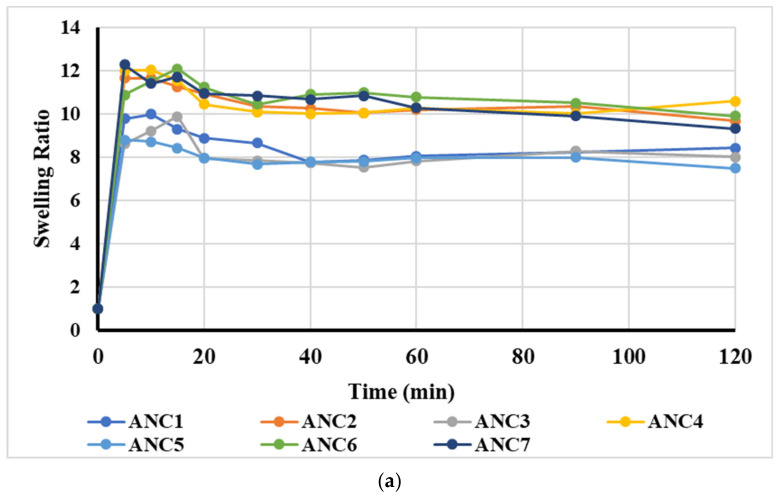

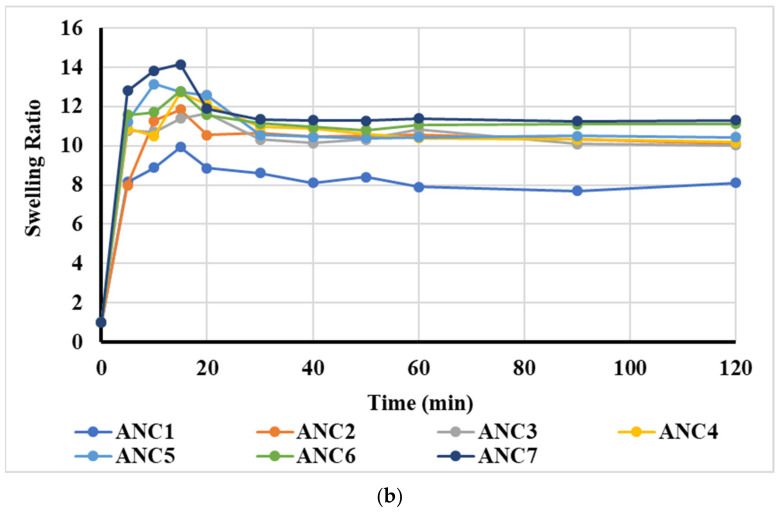
(**a**) The swelling ratio of the developed nanomatrices (ANC1-ANC7) in SGF. (**b**) The swelling ratio of the developed nanomatrices (ANC1-ANC7) in SIF.

The effect of polymer concentration on the swelling ratio was observed in 0.1 N HCl of pH 1.2 and phosphate buffer solution (PBS) of pH 6.8, as shown in Figure 2a,b, and it was seen that when the polymer (chitosan) concentration was increased from 0.3 g to 0.4 g (ANC-1 to ANC-2), the swelling index increased. However, with a further increase in polymer concentration up to 0.5 g (ANC-3), a decrease in the swelling index was seen at both pH conditions (Figure 2a,b). The observed swelling pattern of nanomatrices was due to chitosan, which possesses both the amine and hydroxyl groups capable of forming ionic linkages to form the inter penetrating network, thus, when chitosan is present in an ample amount in the reaction mixture, the swelling ratio of the system is increased, however, upon further increase, the viscosity of the reaction mixture along with the steric effect overcame the bonding potential of the functional groups, resulting in a decreased swelling ratio [46].

The effect of monomer methacrylic acid concentration, i.e., 2 mL, 3 mL, and 4 mL (ANC-2, ANC-4, and ANC-5) were also observed on the swelling ratio. When the concentration of monomer was increased, the swelling index also increased, as shown in Figure 2a,b. The reason behind this increase was possibly the enhanced hydrolysis of the inter penetrating network, which allowed water molecules to penetrate and resulted in an increased swelling ratio, as the carboxylic group of MAA was ionized, while the chitosan NH_3_^+^ charge went back to NH_2_. Under such conditions, chitosan does not form ionic linkages, leading to a decrease in cross-linking density and an increase in swelling capacity [47].

The effect of cross-linker (MBA) concentration, i.e., 2 g, 3.5 g, and 4 g, was also evaluated and it was noticed that an increase in cross-linker concentration resulted in a decreased swelling trend (ANC-2, ANC-6, and ANC-7), as shown in Figure 2a,b [44]. Flory-rehner’s theory of polymer network swelling explains this phenomenon. The swelling behavior of cross-linked nanomatrices was influenced by two opposite forces: one being the re-tractive force of polymeric chain and the other being the thermodynamics of the system. These two forces are in equilibrium to project the swelling trend of nanomatrices. Secondly, more cross-linking points were formed in the system, which caused the swelling ratio to decrease, as shown in Figure 2a,b [48].

### 2.3. Particle Size Analysis

The particle size of cross-linked nanomatrices was determined by zeta sizer (Malvern Zeta-sizer, Worcestershire, UK). The average particle sizes of the developed nanomatrices (ANC-1 to ANC-7) are presented in Table 2. A small values of polydispersity index (PDI), i.e., 0.394, 0.385, 0.390, 0.405, 0.391, and 0.374 and 0.369 of formulation ANC-1 to ANC-7, respectively with 89.9, 91.4, 89.8, 94.7, 96.6, 93.6, and 92.9% intensity, respectively, indicated that the fabricated nanomatrices system has low tendency to form clusters/aggregates. Particle size has an important role in the solubility enhancement, as a reduction in particle size results in an increase of the surface area, which in turn enhances the wettability, swelling, dissolution, and solubility. According to the Ostwald–Freundlich equation, solubility is directly related to the particle size. Similar findings have been reported previously by Badshah and Kifayat et al., who enhanced the solubility of poorly soluble drug chlorthalidone and olanzapine by incorporating the drug into the nano-sized nanomatrices delivery system [25,47].

### 2.4. SEM Analysis

**Figure 3:**
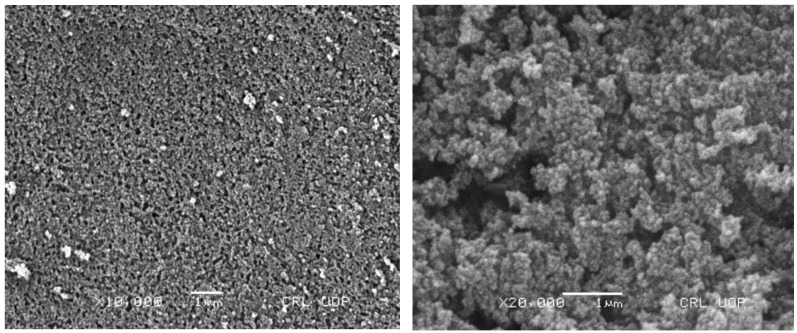
SEM images of nanomatrices formulation at different magnifications.

Developed cross-linked nanomatrices were observed under scanning electron microscope (SEM) for its surface morphological examination and role in the dynamics of the system. Under different resolutions, a porous and fluffy structure of nanomatrices was observed, as shown in Figure 3. Previously, Kifayat et al. also reported a similar observation, which is in agreement with our results, where they prepared nanogels for solubility enhancement of poorly soluble drug meloxicam [48]. Since a porous structure is highly suitable for drug loading and diffusion of the solvent as well as the release of the drug from the system, maximum drug was therefore entrapped in the cross-linked nanomatrices, as shown in the drug entrapment efficiency. The hydrophilicity of the polymer and monomer renders the surface of the cross-linked nanomatrices, being receptive of water molecules, and hence enhanced the influx of water molecules inside the system and made it swell, which relates to the mechanism of the drug release from the system.

### 2.5. Thermal Analysis of Nanomatrices

**Figure 4:**
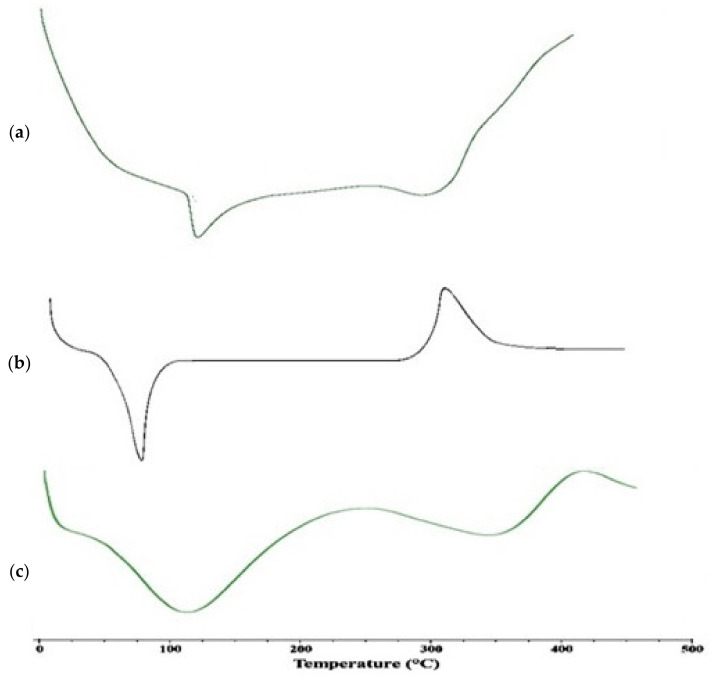
DSC analysis of (**a**) pure drug (SIM), (**b**) polymer (chitosan), and (**c**) SIM-loaded nanomatrices.

Figure 4a–c present the DSC analysis of pure drug (SIM), polymer (Chitosan), and SIM-loaded nanomatrices, respectively. Differential scanning calorimetry refers to the thermal analytical techniques of heat flow through sample and response, and is measured as a function of temperature, i.e., a curve is drawn between the heat flux vs. the temperature. From the DSC thermogram of pure simvastatin, an endothermic peak at 138.8 °C indicated the melting point of the drug. Whereas pure chitosan showed an endothermic peak at 91 °C, which refers to water loss adsorbed by chitosan, another exothermic peak at 317 °C indicated the melting point of the polymer. However, in the nanomatrices formulation, a broader endothermic peak was observed, bearing the polymer and drug peak inside, which indicated the compatibility and stability of the drug–polymer in the system, as shown in the Figure 4 [18].

### 2.6. Powder X-ray Diffraction

**Figure 5:**
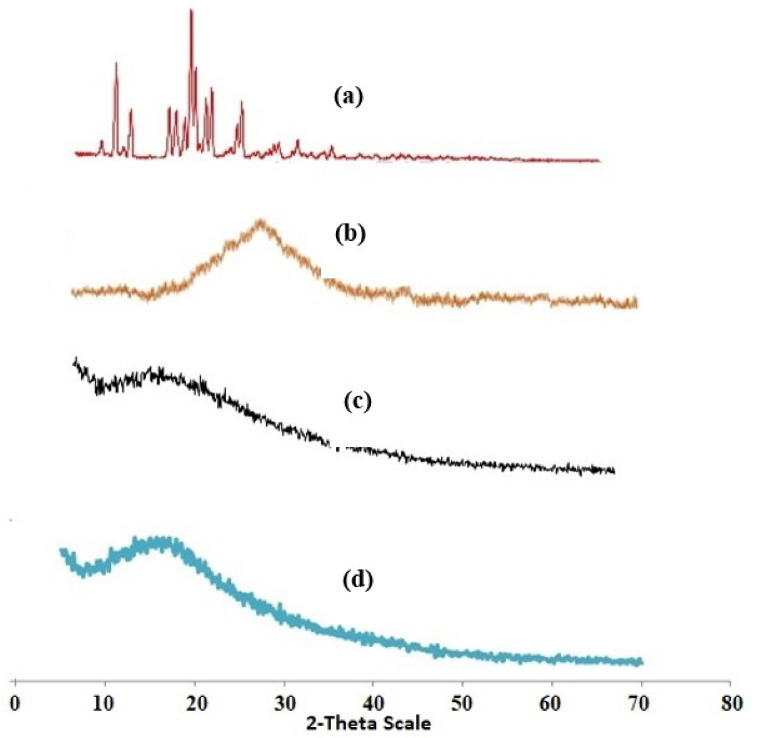
X-ray diffraction patterns of (**a**) pure drug (SIM), (**b**) polymer (chitosan), (**c**) unloaded nanomatrices, and (**d**) SIM-loaded nanomatrices.

Figure 5a–d present the X-ray diffraction patterns of pure drug simvastatin (SIM), polymer (chitosan), un-loaded nanomatrices, and SIM-loaded nanomatrices, respectively. PXRD analysis of the drug (SIM) showed sharp peaks at 9.3°, 10.08°, 14.92°, 15.6°, 17.18°, 17.64°, 18.74°, 19.32°, 21.98°, and 22.5°, which suggested the crystalline nature of the drug [49]. Chitosan showed one broad diffraction peak that was centered at 20.0 °C and very slightly shifted to a higher diffraction angle. The diffractogram of chitosan suggested the semi-crystalline nature of the polymer [50]. PXRD analysis of the unloaded cross-linked nanomatrices suggested a bit more of an amorphous characteristic picture due to the conjugation of polymer with monomer and cross-linker. However, PXRD analysis of drug loaded cross-linked nanomatrices showed a substitution pattern from sharp peaks of the drug to dense peaks, which represented an increase in the pattern of amorphous form. This whole picture suggested that the drug is dispersed in an amorphous form in the polymeric nanomatrices network, which eventually potentiated the solubility characteristics of the drug as well as enhanced its dissolution performance.

### 2.7. Solubilization Efficacy

**Figure 6:**
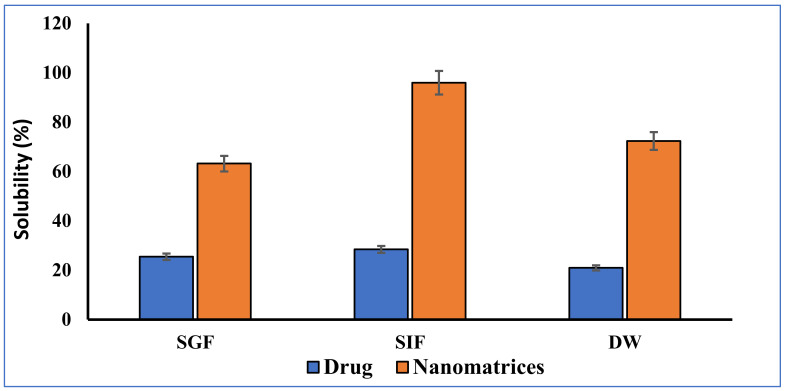
Percentage solubility study of pure drug SIM and drug in nanomatrices at SGF (pH 1.2), SIF (pH 6.8) and DW.

Enhancement of simvastatin solubilization efficacy in de-ionized water (DW), 0.1 N HCl (pH 1.2) (simulated gastric fluid), and phosphate buffer solution (PBS) of pH 6.8 by the prepared optimized nanomatrices (ANC-5) was evaluated and compared to the solubility of the pure drug. Figure 8 presents the solubility enhancement of the pure drug and prepared nanomatrices (ANC-5). It was found that the solubility of poorly soluble drug simvastatin (SIM) was increased up to 2.48-, 3.37-, and 3.13-folds in 0.1 N HCl, phosphate buffer solution (PBS) of pH 6.8 and de-ionized water, respectively, as shown in Figure 8. Solubility was improved due to the hydrophilic and highly amorphous nature of the prepared nanomatrices. Kifayat et al. also reported the solubility enhancement of the poorly soluble drug olanzapine by developing nanomatrices using a different composition of hydrophilic ingredients [29]. Polymer chitosan has already been used previously for solubility enhancement of carvedilol, nifedipine, and curcumin [3,51,52]. Saal et al. enhanced the solubility of drugs by using methacrylic acid copolymer [53]. In another study, Alam et al. reported the solubility enhancement of simvastatin by grafting with methacrylic acid at intestinal pH. The solubility of SIM by nanomatrices has significantly enhanced, as compared to its pure form, as shown in Figure 6 [54].

### 2.8. In Vitro Dissolution Studies

**Figure 7:**
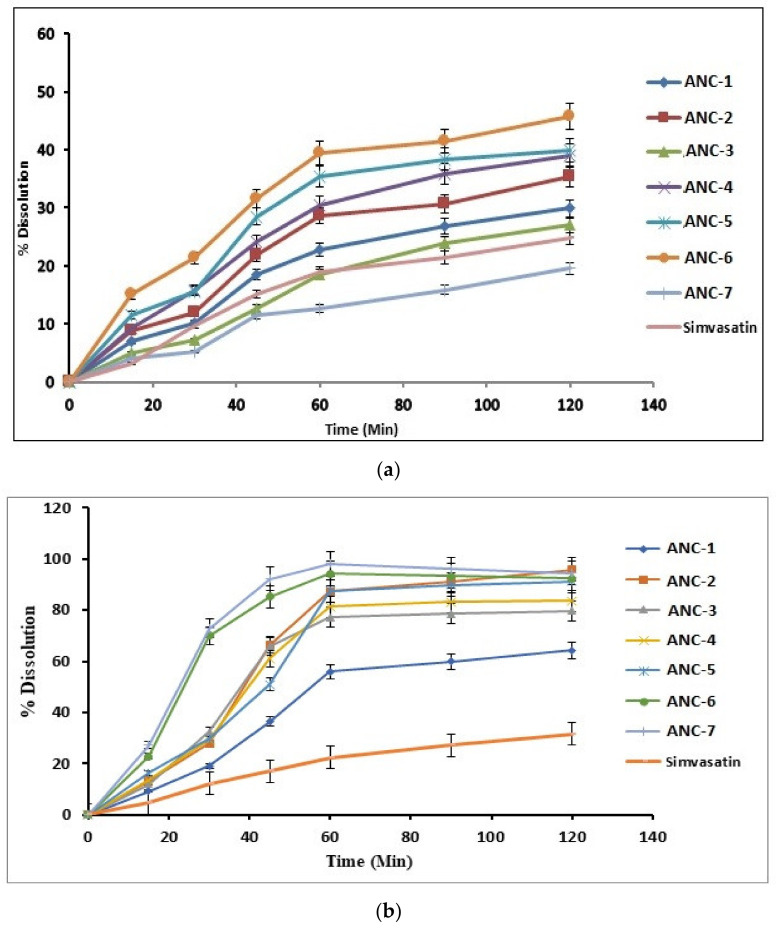

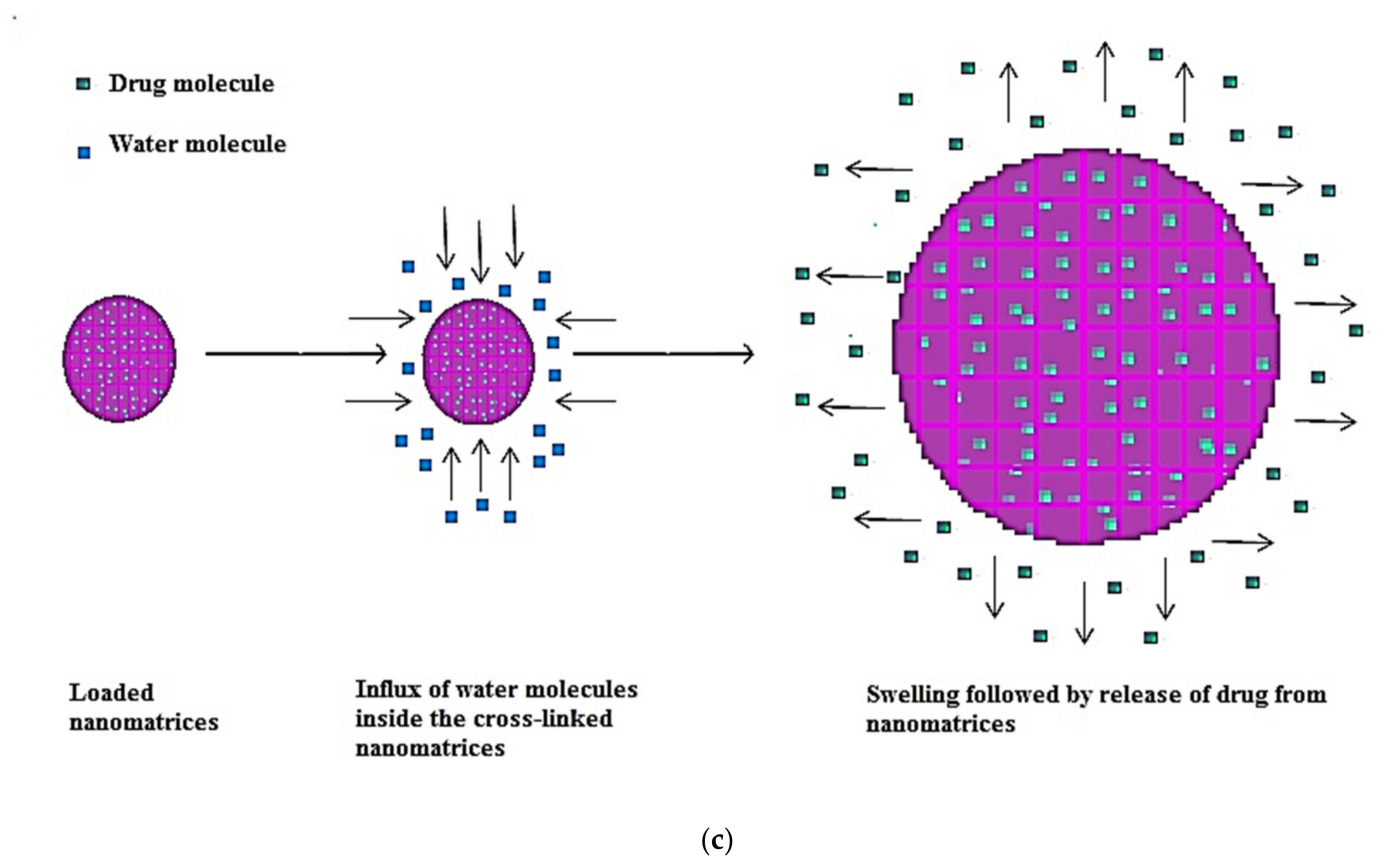
(**a**) Dissolution profile of the developed nanomatrices and pure drug in 0.1 N HCl (simulated gastric fluid) of pH 1.2. (**b**) Dissolution profile of the developed nanomatrices and pure drug in phosphate buffer (simulated intestinal fluid) of pH 6.8. (**c**) Mechanism of drug release from cross-linked nanomatrices.

The in vitro drug release of loaded cross-linked nanomatrices was conducted in 0.1 N HCl of pH 1.2 and phosphate buffer solution (PBS) of pH 6.8 along with a reference product of simvastatin in order to compare the percent drug release. The results of dissolution studies are presented in Figure 7a,b. Simvastatin (SIM) is acidic in nature, so the drug release pattern exceled in PBS of pH 6.8, which might be due to the hydrophilicity of the system, which rendered the drug in an amorphous form. In 0.1 N HCl solution, protonation of the primary amino group of MAA formed NH_3_^+^ and allowed chitosan (polymer) to form a network through ionic linkage with carboxylic group of monomer (methacrylic acid), and thus decreased the swelling capacity as well as drug release observed at lower pH (1.2), as shown in Figure 9a. However, at PBS pH 6.8, the carboxylic group of MAA are ionized, while chitosan the NH_3_^+^ charge goes back to NH_2_. Under such conditions, chitosan does not form ionic linkages, leading to decreased cross-linking density, more influx of water molecules, increased in swelling capacity, and results in a generous drug release of the loaded drug, as shown in Figure 7b. Previously, Shiraishi et al. also enhanced the dissolution rate of several hydrophobic drugs by low molecular weight chitosan [55].

Apart from the pH effect on drug release behavior, the effect of polymer (chitosan), monomer (methacrylic acid), and cross-linker (MBA) concentration were also evaluated. Drug release pattern of all formulations, i.e., ANC-1 to ANC-7, mimics the swelling and drug entrapment efficiency (%) of the system. With an increase in the polymer concentration, i.e., ANC-1 and ANC-2, we observed an increase in the swelling ratio, the drug entrapment efficiency (Table 4) of the system, and eventually the drug release, as shown in Figure 7a,b. A further increase in polymer concentration, i.e., ANC-3 did not increase the drug release, which was due to an increase in the viscosity of the reaction mixture, which halted the swelling and in turn decrease the drug release from the system. Previously, a similar pattern of drug release was also reported by Shamshad et al. [56].

By increasing the monomer concentration, i.e., ANC-2, ANC-4, to ANC-5, we observed an increased drug release pattern, which may be due to the increased hydrolysis of the interpenetrating cross-linked network, which encouraged the penetration of water molecules in the system, and thus enhanced drug release was observed, as shown in Figure 7. A similar trend was reported by Rizvi et al. [34]. An increase in cross-linker (MBA) concentration resulted in a decrease in the drug release pattern, i.e., ANC-2, ANC-6, to ANC-7, which might be due to tight junctions that did not allow the dissolution media to penetrate into the system, as shown in Figure 9. Similar findings were also reported by Minhas et al. [57].

Nanomatrices with a high percent drug entrapment efficiency (%DEE) up to 92.3% ensured the productivity of the preparation procedure and the nanomatrice system’s capacity to entrap the drug. Nanomatrices (ANC-5) were chosen as the best formulation among all based on swelling, sol-gel analysis, drug release, drug loading, and DEE. In an optimized formulation, i.e., ANC-5, the maximum reactive sites of polymer chitosan and monomer methacrylic acid produced by an initiator (BPO) were cross-linked by MBA (cross-linking agent) as a different feed content ratio, which was used for the nanomatrices formulations (ANC-1 to ANC-7), as presented in Table 5. Optimized formulation (ANC-5) was then selected for solubility, FTIR, DSC, and SEM analysis as well as for in vivo toxicity studies.

Nanomatrices are a highly responsive polymeric network that abruptly swell upon contact with aqueous medium due to their highly amorphous nature and porous structure, and the entrapped drug immediately releases into the surrounding media [29]. The release mechanism from cross-linked nanomatrices is shown in Figure 7c.

### 2.9. Fourier Transform Infrared Spectroscopy (FTIR) Analysis

**Figure 8:**
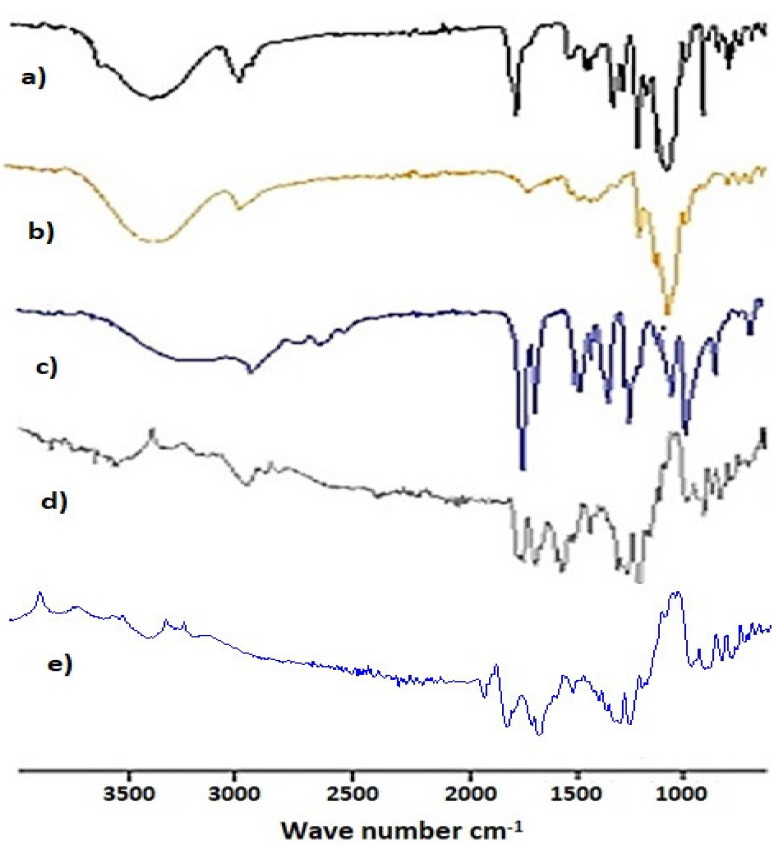
FTIR spectra of (**a**) pure drug (SIM), (**b**) polymer (chitosan), (**c**) monomer (MAA), (**d**) simvastatin loaded nanomatrices, and (**e**) un-loaded nanomatrices.

Figure 8a–e presents the FTIR spectrum of a pure drug simvastatin (SIM), polymer (chitosan), monomer (MAA), simvastatin-loaded, and un-loaded nanomatrices, respectively. FTIR peaks of the pure chitosan were detected at 3291 cm^−1^, which shows an overlapping of –OH and N–H stretching vibrations, the peak at 2874.91 cm^−1^ shows stretching vibrations related to C–H, the peak at 1650 cm^−1^ shows C=O stretching due to the amide group, the peak at 1569 cm^−1^ for N-H bending due to the amide group, and the peak at 1320 cm^−1^ for the C–N stretching amide group. Peaks in the range of 1150 cm^−1^ and 898 cm^−1^ represented C–O and C–O–C symmetrical and asymmetrical stretching vibration in LMW chitosan pyranose ring [58,59]. FTIR spectra of pure simvastatin showed characteristic hydroxyl stretching at 3550 cm^−1^, the peak at 2957 cm^−1^ represents C–H asymmetric stretching vibration, the peak at 1704 cm^−1^ represents ester stretch, and the peak at 1266 cm^−1^ represents lactone carbonyl stretching [18,60]. In the FTIR spectra of MAA, the peak in the range 3200–3500 cm^−1^ represents O–H stretching vibrations due to carboxylic acid. The peak at 2928 cm^−1^ represents C–H stretching due to the methyl group. The peak at 1690 cm^−1^ shows C=O stretching due to the carboxylic group [61]. FTIR spectra of simvastatin and unloaded cross-linked nanomatrices were compared to loaded cross-linked nanomatrices, and it was evident that it is a combination of chitosan, simvastatin, and methacrylic acid with a slight shift in peak positions. The FTIR spectra of loaded cross-linked nanomatrices showed a shift of bands towards 3546 cm^−1^, 1697 cm^−1^, and 1167 cm^−1^, which suggested an intermolecular hydrogen bonding between the –C=O group of simvastatin and the –OH group of chitosan. This interaction predicted a stable amorphous state of simvastatin in the nanomatrices.

### 2.10. Acute Oral Toxicity Studies

**Figure 9:**
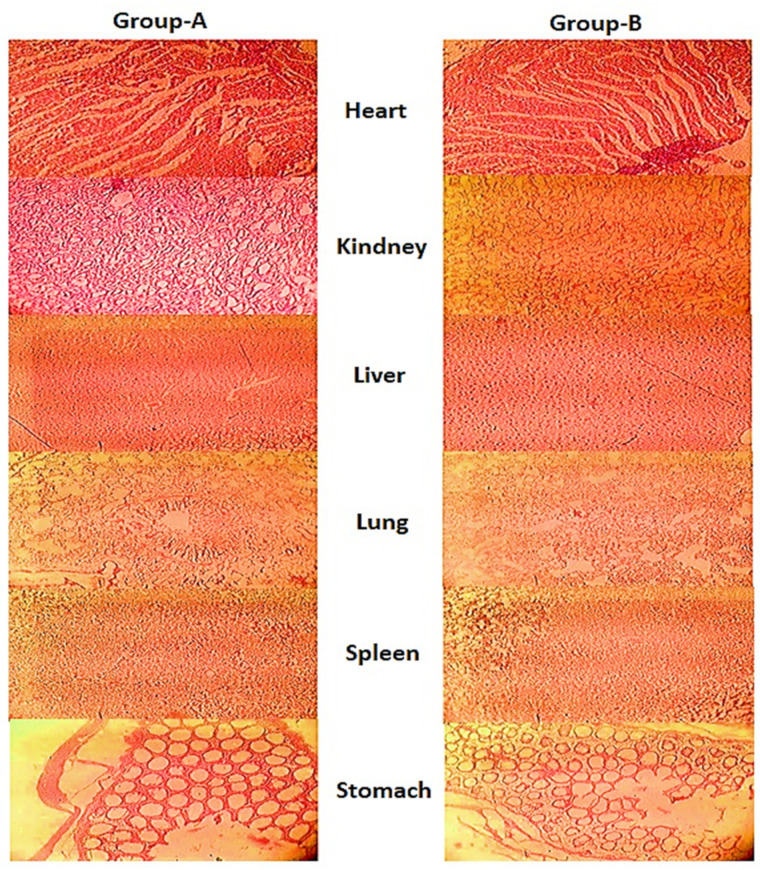
Histopathological tissue examination of different organs of the control group-A and the nanomatrices treated group-B.

To evaluate the safety profile of the developed nanomatrices formulation, an acute oral toxicity study was conducted. Two groups were monitored, where one was controlled and the other was the test group, for a period of 14 days. During the toxicity study, neither toxicity nor mortality were observed in any group. After the 14th day of the study, animal’s heart, lungs, stomach, spleen, kidney and liver were dissected and histopathological examination was carried out, which found no signs of toxicity, as shown in Figure 9.

Histopathological micrographs showed no sign of inflammation in the kidney, the liver showed no sign of inflammation, and intact lining was found for the ducts and hepatocyte. No damage was seen to any part of the lung tissue and alveolar sacs. The cell structure and lining of the spleen were seen as intact. The endothelial lining and mucosa of the stomach were be seen as intact, clear cells with no damage. Both groups were also observed physically during the study period, and showed no sign of general illness, eye irritation, lacrimation, dermal irritation, salivation, convulsions, and hyperactivity. Both the groups responded positive to corneal reflex, alertness, touch response, gripping strength, and alertness. Body weights, water intake and food intake were also not disturbed in any of the group, as presented in Table 3. Moreover, hematological and biochemistry parameters of the test group were comparable to the control group, as shown in Table 4.

## 3. Conclusions

The solubility and dissolution characteristics of the BCS class-II drug were significantly enhanced by nanomatrices drug delivery system, developed by cross-linking hydrophilic components, i.e., chitosan (polymer) and methacrylic acid (MAA) (monomer), via free radical polymerization technique. A fluffy and porous surface morphology was evident from SEM analysis. Crystalline drug was encapsulated in the amorphous nanomatrices as depicted from the PXRD and FTIR spectra. Particle size was in the nano-size range. The formulation was thermally stable. Swelling and in vitro drug release studies were performed in simulated gastric fluid and simulated intestinal fluid and followed the pH-dependent swelling and drug release pattern, i.e., an increase in swelling at SIF eventually increased the drug release in that medium as compared to the pure drug and vice versa. The toxicity study revealed the biocompatible status of the cross-linked nanomatrices.

## 4. Materials and Methods

### 4.1. Materials

Simvastatin was received as a gift from Bio fine pharmaceuticals (PVT) Ltd., Pakistan assay 99.8%. Chitosan LMW 50,000–190,000 Da 75–85% de-acetylated, methacrylic acid (MAA) 99% purity, *N*,*N*′-methylene bisacrylamide (MBA) 99% purity and benzoyl peroxide (BPO) assay 72, 0–77, 0% (m), ethanol 99.8%, and acetone 99.9% were purchased from Sigma Aldrich, St. Louis, MO, USA. Freshly prepared deionized water was acquired from IUB research lab.

### 4.2. Methods

#### 4.2.1. Preparation of Nanomatrices

**Figure 10:**
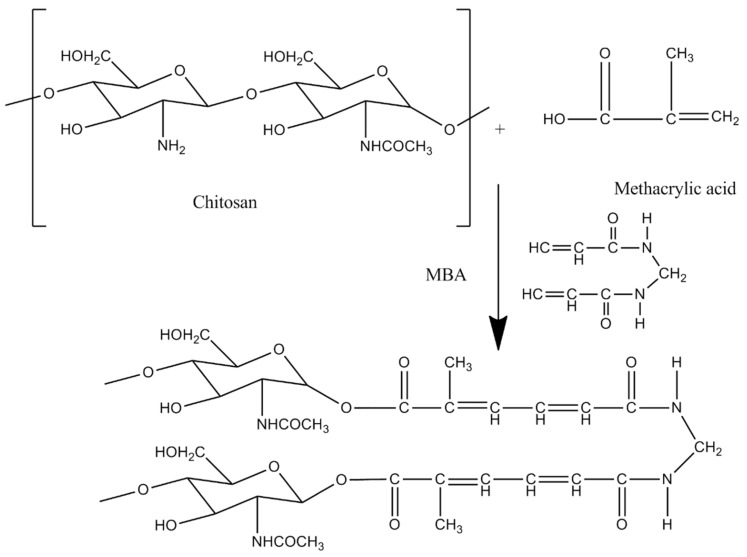
Proposed schematic diagram of the prepared nanomatrices.

Free radical polymerization with subsequent condensation technique was used for the preparation of cross-linked nanomatrices with slight modification of our previously reported technique [25]. For this purpose, initially polymeric solution of chitosan was prepared by dissolving the accurately weighed amount of chitosan in 0.2% *v*/*v* acetic acid solution with continuous stirring. Measured quantity of methacrylic acid was added as monomer to the polymeric solution of chitosan. An initiator benzoyl peroxide was measured accurately and added to the polymer mixture in order to produce the free radicals in the previously prepared polymeric solution. Finally, transparent aqueous solution of cross-linker (MBA) was prepared by solubilizing MBA in a distilled water:ethanol (2:1) mixture and added to the above mixture with continuous stirring. The stirring process was done using a hot plate/magnetic stirrer at 250–300 rpm at 37 °C. The resultant reaction mixture was carefully transferred into a round bottom flask and then reflux condensation was carried out at 85 °C for 4–5 h in order to complete the cross-linking of the reactants. After the specified time, the resultant fluffy mass was recovered, sieved, and dried in an oven at 40 °C until constant mass was obtained [35,36,62]. The proposed chemical structure of the developed nanomatrices is shown in Figure 10. The feed content ratio of excipients are presented in Table 5.

#### 4.2.2. Drug Loading of Nanomatrices

Aqueous suspension of cross-linked nanomatrices was prepared by dispersing the accurately weighed quantity of nanomatrices in water, which was then allowed to swell. Drug solution of simvastatin (2%) was prepared by solubilizing simvastatin in acetone. Drug solution was dispersed in aqueous suspension of cross-linked nanomatrices by continuous stirring, centrifuged, and finally lyophilized.

### 4.3. Characterization

#### 4.3.1. Entrapment Efficiency

Entrapment efficiency of cross-linked nanomatrices was determined by absorption and extraction technique to access the effectivity of the drug loading method. A known weighed amount of cross-linked nanomatrices were crushed and dispersed in methanol with sonication for 1 h. Aliquot was filtered through 0.45-micron pore size filter and dilutions were made accordingly. Dilutions were then analyzed by UV Visible spectrophotometer (Pharma Spec 1700; Shimadzu, Tokyo, Japan) at λ_max_ 238 nm [63]. Equations (1) and (2) were used to determine the percent drug loading (%DL) and entrapment efficiency (%DEE), respectively.

(1) $$\mathrm{Drug}\;\mathrm{loading}\;(\%)=\frac{\mathrm{Entrapped}\;\mathrm{drug}\;\mathrm{in}\;\mathrm{nanomatrices}}{\mathrm{Weight}\;\mathrm{of}\;\mathrm{nanomatrices}}\times 100,$$

(2) $$\mathrm{Entrapment}\;\mathrm{efficiency}\;\left(\%\right)=\frac{\mathrm{Actual}\;\mathrm{drug}\;\mathrm{loading}}{\mathrm{Theoretical}\;\mathrm{drug}\;\mathrm{loading}}\times 100.$$

#### 4.3.2. Solubility Study

A solubility enhancement study of simvastatin was conducted in distilled water, 0.1 N HCl solution of pH 1.2, i.e., simulated gastric fluid (SGF) and phosphate buffer solution (PBS) of pH 6.8 (simulated intestinal fluid), respectively. The drug was added in an excess amount into a known quantity of distilled water and in respective HCl (pH 1.2) and phosphate buffer (pH 6.8) solutions. The respective media were allowed to stir for 24 h. Similarly, further drug dispersions in the distilled water and buffer solutions were prepared and accurately weighed cross-linked optimized nanomatrices (ANC-5) were added to the respective drug solution and shaken in the same manner. Resultant suspensions were filtered, diluted where as necessary and analyzed by UV/visible spectrophotometer at λ_max_ 238 nm.

#### 4.3.3. Swelling Study

Swelling behavior of cross-linked nanomatrices in 0.1 N HCl solution of pH 1.2 and SIF (pH 6.8) were observed by using the previously reported tea bag method [25,64]. Nanomatrices were weighed and packed in empty tea bags, immersed in the respective buffer solutions, and removed after the predetermined time intervals, i.e., 5, 10 15, 20, 25, 30, 40, 50, 60, 90, and 120 min. Afterwards, it was pressed between layers of bloating paper to remove any excess water and was then weighed.

#### 4.3.4. Sol-Gel Analysis

Sol-gel fraction of the formulated cross-linked nanomatrices was determined by processing the weighed amount of cross-linked nanomatrices in distilled water at boiling temperature for 4–5 h in a Soxhlet apparatus. The collected mass was then dried in an oven at 40 degrees Celsius until constant mass was obtained. The dried sample was weighed again. Sol-gel fraction was calculated as follows,

(3) $$\mathrm{Sol}\;\mathrm{fraction}=\frac{\left(W_{0}-W_{t}\right)}{W_{0}}\times 100,$$

(4) Gel fraction=100−Sol fraction,

where W_0_ is the weight of cross-linked nanomatrices before extraction and W_t_ is the weight of dried cross-linked nanomatrices after extraction.

#### 4.3.5. Particle Size Analysis

Particle size analysis was done by Zeta sizer (Malvern instruments). Liquid suspension of cross-linked nanomatrices in methanol was prepared and placed in a disposable clear zeta cell. Average particle size was determined using the differential light scattering (DLS) method. The DLS determined the intensity-weight average diameters of prepared nanomatrices.

#### 4.3.6. Stability Studies

The stability of simvastatin-loaded cross-linked nanomatrices were conducted under ICH guidelines and according to the previously reported method [33]. Cross-linked nanomatrices were loaded in glass vials with closure systems and stored in a stability chamber (Memmert Beschickung, Japan) at 39 ± 2 °C and 75 ± 5% relative humidity. The sampling was carried out during the 0th, 3rd, and 6th month. Physical changes, percent drug entrapment efficiency, FTIR, and solubilization efficiency were all analyzed in the formulations.

#### 4.3.7. Scanning Electron Microscopy

Scanning electron microscopy was used to determine the shape and surface morphology of cross-linked nanomatrices. Samples were mounted on the aluminum slab with double adhesive tape. Gold coating on the stub was done by gold sputter in an argon atmosphere. Different images were resulted in various resolutions from signals generated by the interaction of atoms at different depths within the sample with an electron beam, using JEOL Analytical Scanning Electron Microscope (JSM-6490A, Tokyo, Japan).

#### 4.3.8. Fourier Transform Infrared Spectroscopy

Fourier transform infrared (FTIR) spectroscopy (Bruker, Tensor 27, Bremen, Germany) was used in the range 400–4000 cm^−1^ and IR spectra of the unloaded cross-linked nanomatrices, loaded cross-linked nanomatrices, drug, and polymer were compared for interaction in the functional group and fingerprint region using attenuated total reflectance assembly (ATR-FTIR).

#### 4.3.9. Thermal Analysis

Differential scanning calorimetry (DSC) was used to analyze the thermal stability of the loaded cross-linked nanomatrices, drug, and polymer. The sample was heated under an inert nitrogen atmosphere in the range 25–800 degrees Celsius for DSC.

#### 4.3.10. Powder X-ray Diffraction Analysis

Powdered X-ray diffractometer (PXRD) JEOL JDX 3532, Japan was operated at 5°–60° with 2θ at the rate of 3°/min and the resulting PXRD patterns of loaded cross-linked nanomatrices, unloaded cross-linked nanomatrices, drug, and polymer were evaluated for crystalline or amorphous nature, which grossly affects the solubility.

#### 4.3.11. In Vitro Drug Release Studies

In vitro drug release of drug loaded cross-linked nanomatrices formulations (ANC-1 to ANC-7) were investigated using USP dissolution apparatus-II in 0.1 N HCl solution of pH 1.2 (simulated gastric fluid) and a phosphate buffer of pH 6.8 (simulated intestinal fluid), respectively. The reported tea bag test was used for the drug release studies. A specific quantity of the prepared formulation was placed in the bags and immersed into the respective dissolution medium [65,66]. A drug release study was also carried out for the reference product of simvastatin in order to compare the drug release with the prepared nanomatrices. For this purpose, 500 mL HCl (pH 1.2) and phosphate buffer (pH 6.8) solutions were prepared and the temperature of the system was set at 37 ± 0.5 °C and kept stirring at 50 rpm for 12 h. Five mL aliquots were withdrawn at pre-determined time intervals and replaced with fresh medium, filtered through 0.45 μm pore size filter, and analyzed using an UV spectrophotometer (UV-1601 Shimadzu).

### 4.4. Acute Oral Toxicity Studies

#### 4.4.1. Animal Selection, Housing, and Preparation

Healthy adult female Wistar albino rats (*n* = 5) weighing 210 g ± 20, between 8 and 12 weeks, were obtained from the Animal House of Pharmaceutics Department, The Islamia University of Bahawalpur, Punjab, Pakistan. Before starting the study, its protocol was approved from the Pharmacy Animal Ethics Committee under reference number PAEC/2020/20. Rats were divided into two groups, where one was the control group and the other was the test/treated group. Animals were provided easy access to food and water, housed in ventilated wooden cage in facility where temperature was controlled at 22 ± 3 °C, humidity NLT 30% but NMT 70%, and provided with an artificial 12 h day/night cycle. Rats were acclimatized in a transient room for 5 days to laboratory conditions.

#### 4.4.2. Preparation and Administration of Dose

Acute oral toxicity-fixed dose procedure was followed according to the Organization for Economic Co-operation and Development (OECD) guidelines. Animals were fasted overnight but were given free access to water. The control group was given normal saline as per body weight, i.e., 1 mL/100 g orally and the test group was given a dose of cross-linked nanomatrices 5000 mg/kg weight of rat in the form of suspension by lavage prepared in the normal saline in the same manner as for the control group. After dose administration, food was withheld for 3–4 h. Animals were observed for 14 days [56].

#### 4.4.3. Clinical Observation

All animals were observed for 24 h periodically post dosing with keen attention during the first 4 h and then daily for 14 days. Animals were observed for any changes in behavior pattern, i.e., lethargy, color and texture of skin and fur, eyes, and mucous membranes, autonomic and CNS system, i.e., tremors, convulsions, diarrhea, sleep, and coma, somatic motor activity, i.e., changes in salivation pattern, respiratory, and circulatory system. Food, water intake, and body weight of both groups were calculated.

#### 4.4.4. Hematology and Biochemical Blood Analysis

After 14 days of observation, all rats were sacrificed by cervical dislocation. Blood samples for hematological analysis was done by cardiac puncture from posterior vena-cava and collected in an EDTA tube and further analyzed for erythrocyte, monocyte, neutrophil, lymphocyte, platelet count, hemoglobin, and hematocrit. However, for biochemical analysis, blood was transferred to SST tube and parameters like alkaline phosphatase (ALP), aspartate transaminase (AST), alanine aminotransferase (ALT), creatinine kinase (CK), urea, glucose, serum creatinine, triglycerides, and cholesterol were determined.

#### 4.4.5. Histopathology Macroscopic Observation

All the animals were subjected to gross necropsy. Vital body organs were extricated, weighed, and preserved in formaldehyde solution (10% *v*/*v*). Tissues were sliced and slides were prepared for histopathological microscopic observation.

## Figures and Tables

**Table 1:** Stability studies of simvastatin-loaded cross-linked nanomatrices.

Sr. No.	Parameters	0-Month	3rd Month	6th Month
01	Physical description	Light yellow color	No visible change in color	No visible change in color
02	Entrapment efficiency (%)	92.30 ± 2.11	91.14 ± 2.03	90.32 ± 1.42
03	FTIR analysis	Performed	No major shift in IR spectra	No major shift in IR spectra
04	Solubilization efficiency	Increased	No significant change	No significant change

**Table 2:** Particle size (intensity-weighted average diameters) of the developed nanomatrices.

Sr. No.	Formulation Code	Average Particle Size of Nanomatrices (nm)
01	ANC-1	221.6 ± 11.4
02	ANC-2	224.7 ± 8.1
03	ANC-3	239.5 ± 7.2
04	ANC-4	231.8 ± 8.4
05	ANC-5	227.8 ± 17.8
06	ANC-6	241.6 ± 11.0
07	ANC-7	246.1 ± 12.3

Values are expressed as mean ± SD (*n* = 3).

**Table 3:** Clinical remarks on the acute toxicity study for orally administered nanomatrices.

Observations for Acute Oral Toxicity Study for Control and Nanomatrices Treated Rats		
Parameters	Control Group I	Nanomatrices-Treated Group II
**Water intake per animal per day (mL)**		
Before treatment	31 ± 1.24	32 ± 1.43
Day 1	29 ± 1.09	31 ± 1.18
Day 7	35 ± 0.89	37 ± 1.09
Day 14	39 ± 1.37	40 ± 1.43
**Food intake per animal per day (kg)**		
Before treatment	14 ± 1.17	15 ± 0.96
Day 1	16 ± 1.02	13 ± 1.11
Day 7	15 ± 1.27	14 ± 0.99
Day 14	16 ± 1.07	17 ± 1.15
**Weight of animal (kg)**		
Before treatment	200 ± 0.76	209 ± 1.23
Day 1	204 ± 1.10	213 ± 1.44
Day 7	211 ± 0.98	217 ± 1.11
Day 14	218 ± 1.14	225 ± 1.22
General illness/eye irritation/dermal irritation,	No	No
Lacrimation/salivation,	No	No
Convulsions/hyperactivity	No	No

All values are expressed as mean ± SD (*n* = 3).

**Table 4:** Biochemical blood analysis in the control and test group of rats.

Plasma Biochemical Analysis	Control Group I	Test Group II
Red blood cells 106/µL	8.60 ± 0.67	8.58 ± 0.88
White blood cells 103/µL	4.3 ± 0.23	4.4 ± 0.34
Hemoglobin g/dL	14.38 ± 1.21	14.22 ± 1.11
Hematocrit %	43.31 ± 0.55	43.49 ± 0.74
Monocytes %	2.17 ± 0.49	2.06 ± 0.55
Lymphocytes %	75.02 ± 0.71	75.54 ± 0.68
Neutrophils %	26.5 ± 0.54	26.01 ± 0.39
MCH pg	17.5 ± 0.72	17.9 ± 0.65
MCV fL (µm^3^)	53.4 ± 0.22	53.7 ± 0.26
MCHC g/dL	33.2 ± 0.41	32.8 ± 0.38
**Serum Biochemical Analysis**	**Control Group I**	**Test Group II**
ALT (U/L)	29.82 ± 0.34	30.66 ± 0.38
ALP (U/L)	120.21 ± 0.19	121.32 ± 0.22
AST (U/L)	106.94 ± 0.15	107.32 ± 0.18
Creatinine (mg/dL)	0.65 ± 0.29	0.67 ± 0.31
Urea (mg/dL)	15.51 ± 0.94	15.55 ± 0.87
Cholesterol (mg/dL)	59.58 ± 0.82	59.64 ± 0.88
Triglyceride (mg/dL)	72.59 ± 0.42	72.39 ± 0.50
Glucose (mg/dL)	109.59 ± 0.64	109.70 ± 0.57

All values are expressed as mean ± SD (*n* = 3).

**Table 5:** Composition of chitosan (polymer), MAA (monomer), MBA (cross-linker), BPO (initiator), solvents (water and ethanol), percent drug loading (%DL), and percent drug entrapment efficiency (%DEE) in nanomatrices formulation.

Sr. No.	Formulation Code	Chitosan (g)	MAA (mL)	MBA (g)	BPO (g)	%DL	%DEE
01	ANC-1	0.3	2	3	0.5	74.12 ± 2.03	82.04 ± 1.2
02	ANC-2	0.4	2	3	0.5	78.45 ± 1.58	89.64 ± 1.54
03	ANC-3	0.5	2	3	0.5	81.82 ± 2.35	85.17 ± 2.01
04	ANC-4	0.4	3	3	0.5	86.84 ± 1.49	90.74 ± 1.68
05	ANC-5	0.4	4	3	0.5	90.04 ± 2.13	92.30 ± 2.07
06	ANC-6	0.4	2	3.5	0.5	72.65 ± 1.67	81.22 ± 2.01
07	ANC-7	0.4	2	4	0.5	68.49 ± 2.02	71.40 ± 1.92

## Data Availability

Not applicable.

## References

[1] Chivere V.T., Kondiah P.P.D., Choonara Y.E., Pillay V. (2020). Nanotechnology-based biopolymeric oral delivery platforms for advanced cancer treatment. Cancers.

[2] Prabhu P., Patravale V. (2016). Dissolution enhancement of atorvastatin calcium by co-grinding technique. Drug Deliv. Transl. Res..

[3] Sharma M., Sharma R., Jain D.K., Saraf A. (2019). Enhancement of oral bioavailability of poorly water soluble carvedilol by chitosan nanoparticles: Optimization and pharmacokinetic study. Int. J. Biol. Macromol..

[4] Khalid Q., Ahmad M., Minhas M.U., Batool F., Malik N.S., Rehman M. (2020). Novel β-cyclodextrin nanosponges by chain growth condensation for solubility enhancement of dexibuprofen: Characterization and acute oral toxicity studies. J. Drug Deliv. Sci. Technol..

[5] Jermain S.V., Brough C., Williamsn R.O. (2018). Amorphous solid dispersions and nanocrystal technologies for poorly water-soluble drug delivery—An update. Int. J. Pharm..

[6] Göke K., Lorenz T., Repanas A., Schneider F., Steiner D., Baumann K., Bunjes H., Dietzel A., Finke J.H., Glasmacher B. (2018). Novel strategies for the formulation and processing of poorly water-soluble drugs. Eur. J. Pharm. Biopharm..

[7] Ndlovu S.T., Ullah N., Khan S., Ramharack P., Soliman M., De Matas M., Shahid M., Sohail M., Imran M., Shah S.W.A. (2019). Domperidone nanocrystals with boosted oral bioavailability: Fabrication, evaluation and molecular insight into the polymer-domperidone nanocrystal interaction. Drug Deliv. Transl. Res..

[8] Hua S. (2020). Advances in oral drug delivery for regional targeting in the gastrointestinal tract-Influence of physiological, pathophysiological and pharmaceutical factors. Front. Pharmacol..

[9] Malviya R., Raj S., Fuloria S., Subramaniyan V., Sathasivam K., Kumari U., Meenakshi D.U., Porwal O., Kumar D.H., Singh A. (2021). Evaluation of Antitumor Efficacy of Chitosan-Tamarind Gum Polysaccharide Polyelectrolyte Complex Stabilized Nanoparticles of Simvastatin. Int. J. Nanomed..

[10] Knapik-Kowalczuk J., Chmiel K., Jurkiewicz K., Correia N.T., Sawicki W., Paluch M. (2019). Physical Stability and Viscoelastic Properties of Co-Amorphous Ezetimibe/Simvastatin System. Pharmaceuticals.

[11] Teo R.D., Tieleman D.P. (2021). Modulation of Phospholipid Bilayer Properties by Simvastatin. J. Phys. Chem. B.

[12] Ahmadi M., Amiribc S., Pecic S., Machaj F., Rosik J., Łos M.J., Alizadeh J., Mahdian R., Da Silva Rosa S.C., Schaafsma D. (2020). Pleiotropic effects of statins: A focus on cancer. Biochim. Biophys. Acta (BBA) Mol. Basis Dis..

[13] Duarte J.A., De Barros A.L.B., Leite E.A. (2021). The potential use of simvastatin for cancer treatment: A review. Biomed. Pharmacother..

[14] Rohilla A., Rohilla S., Kumar A., Khan M., Deep A. (2016). Pleiotropic effects of statins: A boulevard to cardioprotection. Arab. J. Chem..

[15] Bedi O., Dhawan V., Sharma P.L., Kumar P. (2016). Pleiotropic effects of statins: New therapeutic targets in drug design. Naunyn-Schmiedebergs Arch. Exp. Pathol. Pharmakol..

[16] Kulhari H., Pooja D., Prajapati S., Chauhan A. (2011). Performance evaluation of PAMAM dendrimer based simvastatin formulations. Int. J. Pharm..

[17] Anwar M., Warsi M.H., Mallick N., Akhter S., Gahoi S., Jain G.K., Talegaonkar S., Ahmad F.J., Khar R.K. (2011). Enhanced bioavailability of nano-sized chitosan–atorvastatin conjugate after oral administration to rats. Eur. J. Pharm. Sci..

[18] Rizvi S.Z.H., Shah F.A., Khan N., Muhammad I., Ali K.H., Ansari M.M., Din F.U., Qureshi O.S., Kim K.-W., Choe Y.-H. (2019). Simvastatin-loaded solid lipid nanoparticles for enhanced anti-hyperlipidemic activity in hyperlipidemia animal model. Int. J. Pharm..

[19] Pandya P., Gattani S., Jain P., Khirwal L., Surana S. (2008). Co-solvent Evaporation Method for Enhancement of Solubility and Dissolution Rate of Poorly Aqueous Soluble Drug Simvastatin: In vitro–In vivo Evaluation. AAPS PharmSciTech.

[20] Tres F., Hall S.D., Mohutsky M.A., Taylor L.S. (2018). Monitoring the Phase Behavior of Supersaturated Solutions of Poorly Water-Soluble Drugs Using Fluorescence Techniques. J. Pharm. Sci..

[21] Liu T., Yu X., Yin H. (2020). Study of Top-down and Bottom-up Approaches by Using Design of Experiment (DoE) to Produce Meloxicam Nanocrystal Capsules. AAPS PharmSciTech.

[22] Khattab W.M., El-Dein E.E.Z., El-Gizawy S.A. (2020). Formulation of lyophilized oily-core poly-Ɛ-caprolactone nanocapsules to improve oral bioavailability of Olmesartan Medoxomil. Drug Dev. Ind. Pharm..

[23] Kasekar N.M., Singh S., Jadhav K.R., Kadam V.J. (2020). BCS Class II drug loaded protein nanoparticles with enhanced oral bioavailability: In vitro evaluation and in vivo pharmacokinetic study in rats. Drug Dev. Ind. Pharm..

[24] Yu H., Lim L.M., Dong B., Hadinoto K. (2019). Proof-of-concept preparation and characterization of dual-drug amorphous nanoparticle complex as fixed-dose combination of poorly soluble drugs. Drug Dev. Ind. Pharm..

[25] Badshah S.F., Akhtar N., Minhas M.U., Khan K.U., Khan S., Abdullah O., Naeem A. (2021). Porous and highly responsive cross-linked β-cyclodextrin based nanomatrices for improvement in drug dissolution and absorption. Life Sci..

[26] Larrea-Wachtendorff D., Del Grosso V., Ferrari G. (2022). Evaluation of the Physical Stability of Starch-Based Hydrogels Produced by High-Pressure Processing (HPP). Gels.

[27] Shahi S., Roghani-Mamaqani H., Talebi S., Mardani H. (2022). Stimuli-responsive destructible polymeric hydrogels based on irreversible covalent bond dissociation. Polym. Chem..

[28] Sakr M.A., Sakthivel K., Hossain T., Shin S.R., Siddiqua S., Kim J., Kim K. (2022). Recent trends in gelatin methacryloyl nanocomposite hydrogels for tissue engineering. J. Biomed. Mater. Res. Part A.

[29] Khan K.U., Minhas M.U., Badshah S.F., Suhail M., Ahmad A., Ijaz S. (2022). Overview of nanoparticulate strategies for solubility enhancement of poorly soluble drugs. Life Sci..

[30] Zia M.A., Sohail M., Minhas M.U., Sarfraz R.M., Khan S., de Matas M., Hussain Z., Abbasi M., Shah S.A., Kousar M. (2020). HEMA based pH-sensitive semi IPN microgels for oral delivery; a rationale approach for ketoprofen. Drug Dev. Ind. Pharm..

[31] Suhail M., Rosenholm J.M., Minhas M.U., Badshah S.F., Naeem A., Khan K.U., Fahad M. (2019). Nanogels as drug-delivery systems: A comprehensive overview. Ther. Deliv..

[32] Kabanov A.V., Vinogradov S.V. (2009). Nanogels as Pharmaceutical Carriers: Finite Networks of Infinite Capabilities. Angew. Chem. Int. Ed..

[33] Asghar S., Akhtar N., Minhas M.U., Khan K.U. (2021). Bi-polymeric Spongy Matrices Through Cross-linking Polymerization: Synthesized and Evaluated for Solubility Enhancement of Acyclovir. AAPS PharmSciTech.

[34] Rizvi S.S.B., Akhtar N., Minhas M.U., Mahmood A., Khan K.U. (2022). Synthesis and Characterization of Carboxymethyl Chitosan Nanosponges with Cyclodextrin Blends for Drug Solubility Improvement. Gels.

[35] Khan K.U., Akhtar N., Minhas M.U. (2020). Poloxamer-407-Co-Poly (2-Acrylamido-2-Methylpropane Sulfonic Acid) Cross-linked Nanogels for Solubility Enhancement of Olanzapine: Synthesis, Characterization, and Toxicity Evaluation. AAPS PharmSciTech.

[36] Khalid Q., Ahmad M., Minhas M.U. (2018). Hydroxypropyl-β-cyclodextrin hybrid nanogels as nano-drug delivery carriers to enhance the solubility of dexibuprofen: Characterization, in vitro release, and acute oral toxicity studies. Adv. Polym. Technol..

[37] Mutalik S., Anju P., Manoj K., Usha A.N. (2008). Enhancement of dissolution rate and bioavailability of aceclofenac: A chitosan-based solvent change approach. Int. J. Pharm..

[38] Negm N.A., Hefni H., Abd-Elaal A.A., Badr E.A., Kana M.T.A. (2020). Advancement on modification of chitosan biopolymer and its potential applications. Int. J. Biol. Macromol..

[39] Geçer A., Yıldız N., Çalımlı A., Turan B. (2010). Trimethyl chitosan nanoparticles enhances dissolution of the poorly water soluble drug Candesartan-Cilexetil. Macromol. Res..

[40] De Moura M.R., Aouada F.A., Mattoso L.H. (2008). Preparation of chitosan nanoparticles using methacrylic acid. J. Colloid Interface Sci..

[41] Wang L., Wang A. (2007). Adsorption characteristics of Congo Red onto the chitosan/montmorillonite nanocomposite. J. Hazard. Mater..

[42] Brazel C.S., Peppas N.A. (1995). Synthesis and Characterization of Thermo- and Chemomechanically Responsive Poly(N-isopropylacrylamide-co-methacrylic acid) Hydrogels. Macromolecules.

[43] Pimpan V., Thothong P. (2006). Synthesis of cassava starch-g-poly(methyl methacrylate) copolymers with benzoyl peroxide as an initiator. J. Appl. Polym. Sci..

[44] Li J., Zhang L., Gu J., Sun Y., Ji X. (2015). Cross-linking of poly (vinyl alcohol) with N, N′-methylene bisacrylamide via a radical reaction to prepare pervaporation membranes. RSC Adv..

[45] Mahmood A., Sharif A., Muhammad F., Sarfraz R.M., Abrar M.A., Qaisar M.N., Anwer N., Amjad M.W., Zaman M. (2019). Development and in vitro evaluation of (β-cyclodextrin-g-methacrylic acid)/Na^+^-montmorillonite nanocomposite hydrogels for controlled delivery of lovastatin. Int. J. Nanomed..

[46] Badakhshanian E., Hemmati K., Ghaemy M. (2016). Enhancement of mechanical properties of nanohydrogels based on natural gum with functionalized multiwall carbon nanotube: Study of swelling and drug release. Polymer.

[47] Khan K.U., Minhas M.U., Badshah S.F., Sohail M., Sarfraz R.M. (2022). β-cyclodextrin modification by cross-linking polymerization as highly porous nanomatrices for olanzapine solubility improvement; synthesis, characterization and bio-compatibility evaluation. J. Drug Deliv. Sci. Technol..

[48] Khan K.U., Minhas M.U., Sohail M., Badshah S.F., Abdullah O., Khan S., Munir A. (2021). Synthesis of PEG-4000-co-poly (AMPS) nanogels by cross-linking polymerization as highly responsive networks for enhancement in meloxicam solubility. Drug Dev. Ind. Pharm..

[49] Faris T.M., Harisa G.I., Alanazi F.K., Samy A.M., Nasr F.A. (2020). Developed simvastatin chitosan nanoparticles co-crosslinked with tripolyphosphate and chondroitin sulfate for ASGPR-mediated targeted HCC delivery with enhanced oral bioavailability. Saudi Pharm. J..

[50] Anand M., Sathyapriya P., Maruthupandy M., Beevi A.H. (2018). Synthesis of chitosan nanoparticles by TPP and their potential mosquito larvicidal application. Front. Lab. Med..

[51] Portero A., Remuñán-López C., Vila-Jato J. (1998). Effect of chitosan and chitosan glutamate enhancing the dissolution properties of the poorly water soluble drug nifedipine. Int. J. Pharm..

[52] Lim L.M., Tran T.-T., Wong J.J.L., Wang D., Cheow W.S., Hadinoto K. (2018). Amorphous ternary nanoparticle complex of curcumin-chitosan-hypromellose exhibiting built-in solubility enhancement and physical stability of curcumin. Colloids Surf. B Biointerfaces.

[53] Saal W., Ross A., Wyttenbach N., Alsenz J., Kuentz M. (2018). Unexpected Solubility Enhancement of Drug Bases in the Presence of a Dimethylaminoethyl Methacrylate Copolymer. Mol. Pharm..

[54] Alam B.M., Aouak T., Alandis N.M., Alam M.M. (2015). Synthesis, characterization, drug solubility enhancement, and drug release study of poly (methacrylic acid-graft-simvastatin). Int. J. Polym. Mater. Polym. Biomater..

[55] Shiraishi S., Arahira M., Imai T., Otagiri M. (1990). Enhancement of dissolution rates of several drugs by low-molecular chitosan and alginate. Chem. Pharm. Bull..

[56] Malik N., Ahmad M., Minhas M., Tulain R., Khalid I., Barkat K., Rashid A. (2020). Toxicological evaluation of xanthan gum based hydrogel formulation in Wistar rats using single dose study. Acta Pol. Pharm.-Drug Res..

[57] Minhas M.U., Ahmad M., Khan K.U., Sohail M., Khalid I. (2020). Functionalized pectin hydrogels by cross-linking with monomer: Synthesis, characterization, drug release and pectinase degradation studies. Polym. Bull..

[58] Rahman N.A., Abu Hanifah S., Mobarak N.N., Su’Ait M.S., Ahmad A., Shyuan L.K., Khoon L.T. (2019). Synthesis and characterizations of o-nitrochitosan based biopolymer electrolyte for electrochemical devices. PLoS ONE.

[59] Zheng X., Yin Y., Jiang W., Xing L., Pu J. (2015). Synthesis and Characterization of Low Molecular Weight Chitosan. BioResources.

[60] Affandi M.M.M., Tripathy M., Majeed A.B.A., Shah S.A.A. (2016). Solubility enhancement of simvastatin by arginine: Thermodynamics, solute–solvent interactions, and spectral analysis. Drug Des. Dev. Ther..

[61] Pardeshi P.M., Mungray A.A. (2019). Photo-polymerization as a new approach to fabricate the active layer of forward osmosis membrane. Sci. Rep..

[62] Lowman A., Morishita M., Kajita M., Nagai T., Peppas N. (1999). Oral delivery of insulin using pH-responsive complexation gels. J. Pharm. Sci..

[63] Rahamathulla M., Gangadharappa H.V., Veerapu G., Hani U., Alhamhoom Y., Alqahtani A., Moin A. (2020). Characterization, Optimization, In Vitro and In Vivo Evaluation of Simvastatin Proliposomes, as a Drug Delivery. AAPS PharmSciTech.

[64] Pourjavadi A., Barzegar S., Zeidabadi F. (2007). Synthesis and properties of biodegradable hydrogels of κ-carrageenan grafted acrylic acid-co-2-acrylamido-2-methylpropanesulfonic acid as candidates for drug delivery systems. React. Funct. Polym..

[65] Mechtcherine V., Snoeck D., Schröfl C., De Belie N., Klemm A.J., Ichimiya K., Moon J., Wyrzykowski M., Lura P., Toropovs N. (2018). Testing superabsorbent polymer (SAP) sorption properties prior to implementation in concrete: Results of a RILEM Round-Robin Test. Mater. Struct..

[66] Lee K.-R., Kim E.-J., Seo S.-W., Choi H.-K. (2008). Effect of poloxamer on the dissolution of felodipine and preparation of controlled release matrix tablets containing felodipine. Arch. Pharm. Res..

